# Selection for predation, not female fecundity, explains sexual size dimorphism in the orchid mantises

**DOI:** 10.1038/srep37753

**Published:** 2016-12-01

**Authors:** Gavin J. Svenson, Sydney K. Brannoch, Henrique M. Rodrigues, James C. O’Hanlon, Frank Wieland

**Affiliations:** 1 Department of Invertebrate Zoology, Cleveland Museum of Natural History, Cleveland, Ohio, United States of America; 2 Department of Biology, Case Western Reserve University, Cleveland, Ohio, United States of America; 3 Department of Biological Sciences, Macquarie University, Sydney, Australia; 4 Pfalzmuseum für Naturkunde - POLLICHIA-Museum, Bad Dürkheim, Germany

## Abstract

Here we reconstruct the evolutionary shift towards floral simulation in orchid mantises and suggest female predatory selection as the likely driving force behind the development of extreme sexual size dimorphism. Through analysis of body size data and phylogenetic modelling of trait evolution, we recovered an ancestral shift towards sexual dimorphisms in both size and appearance in a lineage of flower-associated praying mantises. Sedentary female flower mantises dramatically increased in size prior to a transition from camouflaged, ambush predation to a floral simulation strategy, gaining access to, and visually attracting, a novel resource: large pollinating insects. Male flower mantises, however, remained small and mobile to facilitate mate-finding and reproductive success, consistent with ancestral male life strategy. Although moderate sexual size dimorphisms are common in many arthropod lineages, the predominant explanation is female size increase for increased fecundity. However, sex-dependent selective pressures acting outside of female fecundity have been suggested as mechanisms behind niche dimorphisms. Our hypothesised role of predatory selection acting on females to generate both extreme sexual size dimorphism coupled with niche dimorphism is novel among arthropods.

Dimorphisms in form and size between males and females, common across arthropods^1^, can be driven by sex-specific selective pressures^2^,^3^. In many arthropod groups, such as the golden orb web-building spider *Nephila clavipes* (Linnaeus, 1767), females have larger bodies to increase fecundity while males remain small for mobility during mate-finding^4^,^5^. Large insect females are typically mobility-limited, making their survival dependent on camouflage and defensive displays^6^. These patterns hold true for most of the predatory praying mantises (Insecta, Mantodea)^6^, which exhibit sexual dimorphism in features of camouflage and body size^7^, with slightly smaller, more flight capable males that track females using olfactory and visual cues^8^. Interestingly, the vast majority of these sexually dimorphic mantis species retain structurally similar sexes that exploit the same ecological niche. However, a lineage of highly camouflaged mantises includes two unusually conspicuous species, the orchid mantises, which appear to have abandoned camouflage colouration and exhibit pronounced sexual size dimorphism (SSD) while also exhibiting sex-dependent cryptic features^8^. Investigating the mechanisms behind the uniqueness of the orchid mantises will advance our understanding of how arthropod sexual dimorphism influences the evolution of morphology and life strategy.

Praying mantises of the Hymenopodini tribe include fascinating examples of disruptive camouflage through the use of colour patterns and cryptic structures to blend with vegetation and disguise body profiles for predator avoidance^9^. All species in the tribe exhibit variously sized cuticular leg expansions appearing as foliaceous lobes, while most are patterned with contrasting green, white, and brown colouration^8^,^10^. Many Hymenopodini species have been suggested to have some association with flowers, residing on the inflorescences themselves^8^,^11^. However, the orchid mantis *Hymenopus coronatus* (Olivier, 1792) (referred hereafter as *Hymenopus*) and the yellow orchid mantis *Helvia cardinalis* Stål, 1877 (hereafter *Helvia*) are unique among the tribe with highly conspicuous monochromatic colouration and extremely enlarged foliaceous lobes on the middle and hind legs that resemble flower petals^8^. In addition, these two genera, together with the banded flower mantis, *Theopropus elegans* (Westwood, 1832) (hereafter *Theopropus*), appear to have unusually pronounced SSD^8^,^12^ (Fig. 1). *Hymenopus* male and female nymphs were shown to be floral simulators, resembling flowers throughout post-embryonic development in order to attract and prey on insect pollinators^8^,^13^. Unlike their namesake suggests, *Hymenopus* nymphs do not live on or maintain a close association with orchids or any particular flowering plant, but are found residing on green vegetation^14^,^15^. *Hymenopus’* floral simulation alone is so convincing that they attract more insect pollinators than sympatric flowers^13^. Amazingly, nymphs of *Hymenopus* even emit allelochemicals that mimic intraspecific pheromone communication of the oriental honeybee *Apis cerana* Fabricius, 1793 as a deceptive predatory tactic^16^. Although adult female *Hymenopus* and *Helvia* ecologies have not been researched, the continued expression of the morphological pollinator attracting features^8^ and preferential feeding on pollinating insects^16^ indicates that they conserve the strategy.

Here we combine a comprehensive phylogeny of Hymenopodidae^8^ with body size measurement data to investigate the taxonomic distribution and origins of sexual dimorphisms in a highly camouflaged group of praying mantises. For our analysis, we reconstructed a time-calibrated phylogeny based on a representative taxon sampling from within Hymenopodidae (Supplementary Table S1), an extensive molecular and morphological dataset^8^, and previously used fossil data and divergence times^17^. We collected measurement data from 129 exemplars (Supplementary Table S2) and calculated female over male SSD ratios for pronotum length and total proportional femoral leg lobe area using average values (Supplementary Tables S3 and S4). We performed Kruskal-Wallis tests on taxon groupings for measurement traits within the morphologically similar Hymenopodini as well as for body scale controlled ratios on a broad selection of taxa from the Hymenopodinae, Acromantinae, and Oxypilinae (HAO group). Group comparisons targeted observed size and ratio differences between the taxa with pronounced SSD including *Theopropus*, *Hymenopus*, and *Helvia* (THH group) and other taxa within Hymenopodini as well as a broader sample of HAO taxa. We tested for sex-dependent contribution to SSD through phylogenetic correlation using sex-based size vs. SSD ratio measures. To test for evolutionary rate shifts in sexually dimorphic features, we modelled trait evolution using the time-calibrated phylogeny to calculate the best set of evolutionary rate shifts given our data.

Using this camouflaged lineage of mantises, we demonstrate for the first time in an arthropod lineage that sex-dependent selective pressures acting on female predatory success rather than reproductive success drove extreme SSD through female gigantism. Utilising a recent wealth of published behavioral studies and morphological observations, we hypothesise the likely evolutionary scenario that gave rise to the remarkable floral simulating orchid mantises based on patterns found.

## Results

### Sexual size dimorphism

THH males were significantly smaller (*p* = 4.3e^−^3; Fig. 2a) and THH females were significantly larger (*p* = 1.2e^−^5; Fig. 2a) compared to males and females of other Hymenopodini, respectively (Supplementary Table S5). We found that SSD was on average 1.7 times greater in THH taxa compared to HAO taxa (*p* = 5.4e^−^3; Supplementary Table S6), which was driven by small THH males (0.87 times smaller) and large females (1.5 times larger). We recovered a well-supported rate shift (BF = 483) for increased SSD on the branch leading to the THH clade with rates increasing with time (Branch 1, Fig. 3a). Female size was correlated significantly with SSD (*p* = 1.1e^−^3) while male size was not (*p* = 0.21), suggesting female gigantism as the cause of the shift towards increased SSD (Supplementary Table S7). Although there is an average background SSD ratio of 1.28 across non-THH Hymenopodinae, the significant SSD increase in the THH clade suggests a shift of ecological strategy.

### Evolution of floral simulation

**Figure 1:**
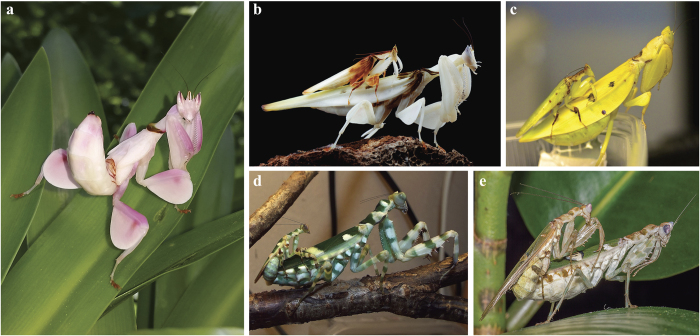
Floral and disruptive camouflage in nymphal and adult Hymenopodini with varying sexual size dimorphism (SSD) of males and females. *Hymenopus* (**a**) female nymph with monochromatic colouration (photograph by Matthew Nochisaki), (**b**) mating pair with pronounced SSD and monochromatic colouration (photograph by Jason Zhu). (**c**) *Helvia* mating pair with pronounced SSD and monochromatic colouration (photograph by Adrian Kozakiewicz). (**d**) *Theopropus* with pronounced SSD, but patterned, disruptive camouflage in both male and female (photograph by Stefan Engelhardt). (**e**) *Creobroter* sp. mating pair with low SSD and patterned colouration in both male and female (photograph by Andrew Mitchell).

**Figure 2:**
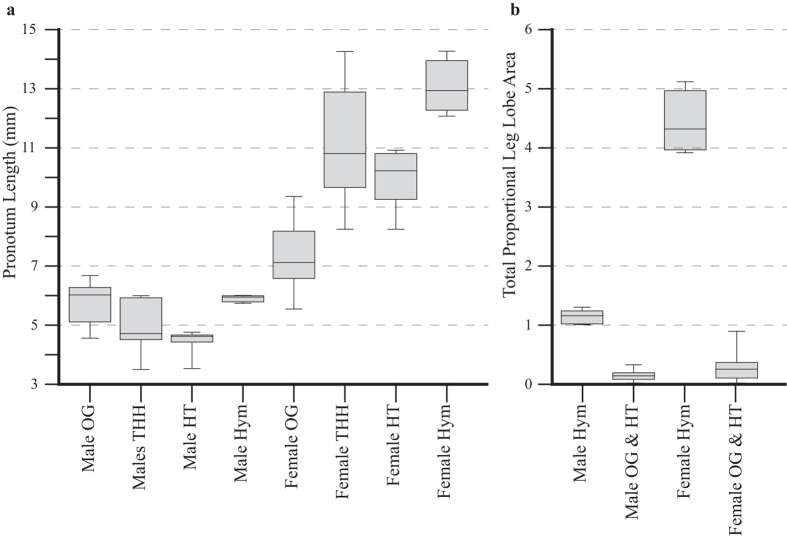
Box plots of pronotum lengths and proportional leg lobe area. (**a**) Males of THH are not significantly different than other Hymenopodini taxa (OG). Treating *Hymenopus* (Hym) separately, *Helvia* and *Theopropus* together (HT) are significantly smaller. Females of Hymenopodini are larger than males across the tribe, but THH females alone are significantly larger than OG females and much larger than their male conspecifics. Treating *Hymenopus* separately demonstrates that females of the genus are the largest in the tribe. (**b**) Total proportional leg lobe area of males and females demonstrates that *Hymenopus* is significantly larger than all other taxa in the tribe.

**Figure 3:**
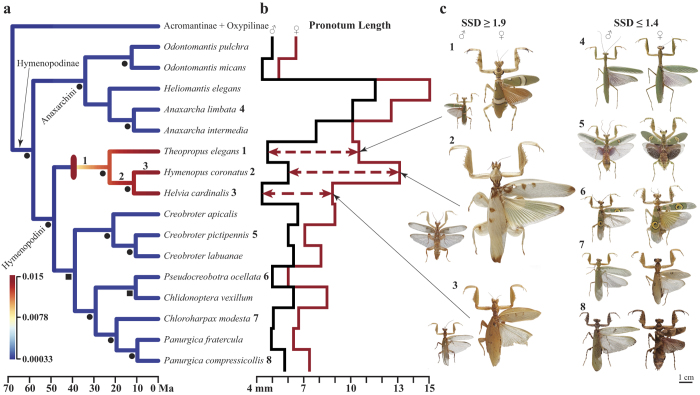
Sexual size dimorphism within Hymenopodinae. (**a**) Time calibrated phylogeny of Hymenopodinae with branch colours according to rates of SSD change (heat map in bottom left indicates rate values). A highly supported rate shift for increased SSD (red ellipse) indicated on branch 1 with shifts for increased proportional femoral leg lobe area on branches 2 and 3. Posterior probabilities (PP) greater than 99 (black circles) and below 75 (black squares) indicate support. Numbers adjacent to species name indicate corresponding photograph to the right. (**b**) Step plot of pronotum lengths for males (black) and females (red) with dashed horizontal lines highlighting taxa with SSD greater than 1.9, which correspond to the three species photographed to the right. (**c**) Scaled images of Hymenopodinae (numbers indicate species on phylogeny) for males and females with SSD ≥1.9 and ≤1.4 (specimen photographs by Rick Wherley).

The initial reason for THH female gigantism is not known, but the remarkable ecological strategy of *Hymenopus* and *Helvia* suggests predatory success could be driving female phenotypic evolution. *Theopropus*, the first branch of the THH clade, is coloured like other Hymenopodini with disruptive camouflage (Fig. 1d,e), but its floral association suggests that the ancestor of THH mantises could have been under selection for a large body to gain predatory advantage over pollinating insects. Small to medium sized pollinators have been shown to change their behaviour to avoid flowers that present signs of predators^18^. However, large pollinators, otherwise protected by their size, maintain their typical foraging behaviour, being an unavailable resource for most flower-associated predators^19^. Selection for a larger body size in the ancestor of THH mantises allowed exploitation of these large pollinators as a novel food resource. Although it is possible that other selective pressures may have contributed to female size increase (e.g., increased fecundity^20^,^21^ or reduced intra-specific competition^1^), the subsequent evolutionary changes within the clade all point to predation as the dominant force shaping female ecology. These factors, floral association and predation on pollinators, likely set the stage for a subsequent transition from camouflaged ambush predation to female floral simulation for prey attraction. After the split with *Theopropus*, human-perceived white/yellow monochromatic colouration originated in *Hymenopus* + *Helvia* (Fig. 3a, Branch 2). White/yellow colouration is attractive to insect pollinators and contrasts against green vegetation irrespective of object shape and symmetry^17^,^22^, with larger surface areas attracting more pollinators^23^. Reflectance spectra collected on the white cuticle of *Hymenopus* demonstrated that the mantis’ colouration is indistinguishable from that of sympatric flowers^13^. We also found a positive rate shift (Fig. 3a, Branch 2, BF = 22) for an increase in the relative size of femoral leg lobes during the origin of *Hymenopus* + *Helvia* (Fig. 3a), which was likely a visual enhancement to attract pollinators. *Hymenopus* females appear to have amplified these enhancements through substantial body and lobe size increases (Fig. 2). Since pollinators are attracted to colours that contrast against vegetation (*e.g.*, human-perceived white, yellow, pink) regardless of shape, the orchid mantises appear to have converged upon the visual space of flowers to dupe potential predators^24^. A large contrasting visual signal would not only attract insect pollinators but also present as an obvious prey item to vertebrate predators. Appearing as a flower in both colour and shape maintains a conspicuous visual signal while masquerading as a flower to would-be predators, which can be considered to be dual-channel crypsis.

### Sex-dependent selective pressures

Adult THH male phenotypic evolution appears to be dominated by reproductive success. Their smaller body size (Fig. 2a), leg lobes (Fig. 2b), and more cryptic colouration (Fig. 1b,c) all point to a life of predator avoidance, mate-finding, and ambush predation, which is consistent with most other praying mantis males^25^. Male *Hymenopus* reach adulthood faster than females^26^, exiting nymphal floral simulation at a much smaller size, indicating adult male ecology is not governed by the same factors as females. Male colouration in *Hymenopus* and *Helvia* includes some monochromatic colouration, but with translucent forewings and disruptive brown patches or stripes on the pronotum, legs, and leg lobes (Fig. 3c). We also recovered a positive rate shift towards larger leg lobes in *Hymenopus* males (Fig. 3a, Branch 3, BF = 15), but their lobes are ~4 times smaller than conspecific females (*p* = 0.014; Fig. 2b). Considering adult male ecology, we would expect them to appear like other camouflaged Hymenopodini males, but their genetic correlation with females may limit disruptive camouflage^27^.

### Extreme limits of sexual size dimorphism

The transition from dwarf THH males to larger males of *Hymenopus* (*p* = 4.5e^−^3) was likely caused by female size increase (Fig. 2a) since SSD remained consistent within the clade (Supplementary Table S4). If one sex becomes too small, constraints on mating mechanics could prevent successful copulation^28^. Though small male size would probably benefit *Hymenopus*, the concordant increase in size suggests that the THH clade may be at the extreme of SSD for praying mantises. To our knowledge, no other praying mantis species exhibits female size greater than twice that of males.

## Discussion

Arthropod SSD is typically linked to female fecundity; generally speaking, the larger the female, the greater the number of offspring^29^. Here, we demonstrate that SSD has been driven by female gigantism in response to selective pressures imposed by predatory success rather than fecundity. Based on ancestral floral associations in the tribe, female gigantism would have improved access to not only a variety of prey, but to a steady source of pollinating insects^18^. The resulting SSD may have alleviated impediments to the evolution of floral simulation by freeing the sexes from shared ecologies^2^, which allowed highly unique female morphology to evolve in a way that would have otherwise harmed male mating success. Although ecological factors influencing sexual dimorphisms and sex-dependent niche exploitation^2^ have been documented in vertebrate groups, the sexes typically partition niches while still sharing similar feeding strategies^30^,^31^. Orchid mantises represent a rare system where sexual dimorphism extends beyond appearance and niche specialisation to the mechanisms a predator uses to encounter their prey. Adult orchid mantis males and females exhibit fundamentally different predatory strategies with males relying on an ancestral ambush predatory strategy and females using aggressive crypsis (Peckhamian mimicry)^32^.

Many spiders also exhibit sex-dependent hunting strategies. For example, female bolas spiders (e.g., *Mastophora* spp.) lure prey to pheromone laced traps while males retain juvenile hunting tactics^33^,^34^. Certain female orb-weaving spiders (e.g., *Gasteracantha fornicata* Fabricius, 1775) are even conspicuously coloured to attract prey to their webs^35^, which is remarkably similar to the strategy employed by *Hymenopus*. Although these arachnid examples are loosely similar to orchid mantises, extreme SSD in arachnids is more common in comparison and often is an ancestral state in lineages that include species with highly derived sex-dependent hunting strategies^36^. The repeated origin of extreme SSD across arachnids suggests multiple evolutionary pathways, but the underlying causes remain unclear^36^. Arachnid SSD is typically thought to be caused by female investment in fecundity through size increase^4^,^20^,^21^,^36^, but it has also resulted from male size selection via limitations to male locomotion and silk use (i.e., Gravity Hypothesis^37^ and Bridging Gravity Hypothesis^38^) or selective mortality favouring smaller individuals^4^. These scenarios indicate arachnid lineages with selective pressures extending beyond female fecundity. However, physical limitations acting on male praying mantis size extending beyond predator avoidance and mate-finding are unknown, although this does not eliminate the possibility that male ecology is under selection from undocumented factors.

The phylogenetic reconstruction we employed to investigate the evolutionary transitions leading to increased SSD and floral simulation links the niche dimorphism of orchid mantises to predatory selective pressures on females. This system, to the best of our knowledge, is the first case of predatory selective pressure, not female fecundity, driving the evolution of SSD in arthropods. Although certain arachnid groups do exhibit extreme SSD^4^,^5^ or niche dimorphisms^39^,^40^, floral simulating praying mantises exhibit both, which is novel among arthropods. Further, predatory selective pressure driving arthropod SSD has not been documented and begets future studies in groups with prevalent SSD, such as arachnids, to determine whether the traditionally accepted female fecundity hypothesis is the dominant driving force behind the evolution of SSD.

## Methods

### Taxon Sampling

To address evolutionary patterns within the Hymenopodini flower mantises we followed the newly revised classification system^8^ and gathered 11 species that are representative of all eight described Hymenopodini genera as well as five species from the sister-group tribe Anaxarchini (Supplementary Table S1). We also included a broader sampling from Hymenopodidae with seven species of Acromantinae and four species of Oxypilinae as well as more distantly related representatives from the Hymenopodidae family^8^ including two species of Phyllocraniinae, three species of Phyllothelyinae, and one species of Sibyllinae. We included 12 outgroup taxa from Empusidae and a broader diversity of mantises to reconstruct the phylogeny. Our sampled ingroup taxa are adequate morphological representatives of each genus since praying mantises are typically homogeneous morphologically within genera^7^.

We captured full habitus images of males and females for reference species using a Passport Storm© system (Visionary Digital™, 2012), which includes a Stackshot z-stepper, a Canon 5D SLR, macro lenses (50 mm, 100 mm, and MP-E 65 mm), three Speedlight 580EX II flash units, and an associated computer running Canon utility and Adobe Lightroom 3.6 software. The z-stepper was controlled through Zerene Stacker 1.04 and images were processed using the P-Max protocol. Images were processed in Adobe Photoshop CS6 Extended to adjust levels, contrast, exposure, sharpness, and add scale bars (1 cm). Minor adjustments were made using the stamp tool to correct background aberrations and to remove distracting debris. Plates were constructed using Adobe Illustrator CS6.

### Measurements

We collected measurements from 129 specimens (75 Male, 54 Female) from the Hymenopodinae, Acromantinae, and Oxypilinae (Supplementary Table S2) for body length, pronotum length, meso- and metafemoral ventral apical femoral lobe area, and meso- and metafemoral length. We used a Leica M165C stereomicroscope with an IC80 HD coaxial video camera using the live measurements module of the Leica Application Suite. Body length was measured using the linear tool from the central ocelli to posterior tip of the abdomen. Pronotum length was measured using the linear tool from anterior margin to posterior margin. Femora were measured using the linear tool from the most proximal margin abutting the trochanter to the distal side of the terminal spine insertion site. Lobe area measurements were performed using the polygon tool with points selected along the margins with enough resolution to ensure demarcated line follows lobe shape. Measurements were collected from multiple specimens within each taxon and sex when possible. Limitations for taxon sampling resulted from rarity of taxa in museums.

Body length across conspecific specimens can vary considerably due to head position during preservation relative to the body as well as abdominal conditions. Depending on food contents for males and females, egg content for females, and hydration levels, the abdomen can vary in length irrespective of the overall size of the specimen. Comparatively, the pronotum is a rigid structure that does not vary due to these factors. We tested the accuracy of body length compared to pronotum length and found the pronotum to recover an average coefficient of variation (CV) ~1.4 times less than body length for both males and females. Specifically, the average CV for male pronotum length was 0.0422 while the average CV for male body length was 0.0589; the average CV for female pronotum length was 0.0432 while the average CV for female body length was 0.0625. Therefore, we used pronotum length as the most accurate assessment of comparative specimen size in our analyses.

The area of apical leg lobes on meso- and metathoracic femora were divided by their respective femoral lengths to control for scaling differences across taxa. These ratios were summed between a set of the meso- and metathoracic legs to produce the total proportional leg lobe area for each taxon within Hymenopodini. This value provides a single measure for the relative size of the leg lobes relative to the size of the specimen. Sexual size dimorphism (SSD) was captured for body length, pronotum length, and total proportional femoral leg lobe area by dividing average measurement values for females by average values for conspecific males.

Elongation in the pronotum relative to the overall body length varies considerably across Hymenopodidae and outgroup taxa^8^. Within the focal Hymenopodini tribe the pronotum is an average of 21.6% and 23.6% of the total body length for males and females, respectively. However, taxa within Anaxarchini and Acromantinae exhibit elongated pronota that are an average of 31.2% and 30.8% of the total body length in males and females, respectively. Therefore, to avoid influence from differences in body scaling on statistical and evolutionary rate shift analyses we pruned our taxon sampling. For length measurements we restricted sampling to only Hymenopodini taxa to ensure compatible morphologies and similar relative component scaling levels (Supplementary Table S3). We used the monophyletic clade including Hymenopodinae, Acromantinae, and Oxypilinae to investigate ratio data, which controls for size scaling problems and broadens taxon representation (Supplementary Table S4).

### Phylogenetic analysis

We performed mixed model Bayesian analysis using a combined molecular and morphological dataset^8^ for our taxon sampling (see Supplementary Table S1 for accession numbers) using MrBayes version 3.2.1^41^,^42^,^43^. The ten gene fragments including 12 S rRNA, 16 S rRNA, 18 S rRNA, 28 S rRNA, Cytochrome Oxidase I (COI), Cytochrome Oxidase II (COII), NADH dehydrogenase subunit 4 (ND4), Histone 3 (H3), Histone 2 A (H2A) and Wingless (wnt-1) were aligned with mafft v7^44^,^45^ and ribosomal genes (12 S, 16 S, 18 S and 28 S) were then piped through GBlocks v0.91b^46^ to remove hyper-variable regions (parameters: allowed gaps = half, minimum length of a block = 5, maximum number of contiguous non-conserved positions = 12). For phylogenetic analyses, the concatenated dataset was divided into eight partitions: mitochondrial codon positions 1, 2 and 3, nuclear codon positions 1, 2 and 3, mitochondrial ribosomal loci, and nuclear ribosomal loci. A ninth partition was comprised of the 124 morphological characters^8^ (characters and descriptions can be accessed through DRYAD DOI:10.5061/dryad.rk07d) and modelled under the mk1 model of Lewis^47^. We modelled all molecular partitions with the GTR + Gamma model (six gamma categories). We implemented two runs with four chains each for 30 million generations. Each run was started from a random tree and subsequently monitored for convergence using the program Tracer ver. 1.5^48^. All sampled generations (every 1000) prior to stationarity were discarded (burn-in). The trees sampled from the stationarity distribution were summarised as a 50% majority rule consensus tree to find posterior probabilities (PP)^49^,^50^. FigTree v1.4.2^51^ was used to visualise trees and export for figure layout (Supplementary Fig. S1).

### Divergence time estimation

We estimated a time calibrated tree for character rate shift analysis using MrBayes with an independent gamma rate (IGR) modelled clock^52^. We used a normal prior probability distribution (0.01, 0.005) for the rate of the Poisson process. We implemented an exponential prior (10) on the variance of the gamma distribution for branch lengths. Due to the rarity of fossils representative of extant Mantodea lineages, we were only able to include a single internal calibration (offset lognormal 60, 60.03, 0.24) in our analysis based on *Prochaeradodis enigmaticus* Piton, 1940 (shale, 60 Ma), which has been placed in the Mantidae subfamily Choeradodinae^53^. We calibrated the root age (uniform, 113.90–137.46) based on prior results^54^, which have recently been corroborated^55^. All sampled generations (every 1000) prior to stationarity were discarded (burn-in). We calculated a fully resolved posterior topology for analysing character evolution, which recovers nodes with the greatest occurrence (possibly less than 0.5).

### Evolutionary rate shift analysis

We conducted Bayesian analysis of macroevolutionary mixtures (BAMM)^56^ on our trait data including pronotum length and total proportional femoral leg lobe area for both males and females using the pruned Hymenopodini-only time-calibrated phylogeny. Averages for taxa were used as terminal data points (Supplementary Table S3). In addition, we investigated rate shifts for sexual size dimorphism using ratios calculated for each taxon from averages from males and females for the pronotum length and total proportional femoral leg lobe area (Supplementary Table S4) using the time-calibrated phylogeny pruned to include Hymenopodinae, Acromantinae, and Oxypilinae taxa.

We used the BAMMtools package^57^ in R (R Development Core Team, 2008) with the function setBAMMpriors to adjust the priors for betaInitPrior (initial phenotypic evolutionary rate associated with regimes) and betaShiftPrior (Parameter (standard deviation) of the prior (normal) on the rate-change parameter for non-root events) to ensure scaling of our tree and the associated tip data would not impact the analysis. These priors were input into the trait control file and implemented in BAMM with associated tip data for 200 million generations with a write frequency every 1000 generations. The evolutionary rate of the trait was calculated under a Brownian motion process, which allowed for independence between parent rates and downstream rates of evolution^58^. We processed event data to determine the posterior distribution of rate shift models (Supplementary Table S8) and the Bayes factor support for each recovered model in the distribution compared to the null (zero rate shifts) using the BAMMtools package in R (Supplementary Table S9). We calculated the *maximum a posteriori* (MAP) probability, which is the overall best rate set of rate shifts given our data as well as the Bayes factor support for these shifts on likely branches recovered by the analysis.

### Statistical analysis

Pronotum lengths and total proportional leg lobe area was plotted using PAleontological STatistics (PAST) Version 3.10^59^ as box plots. The group *Hymenopus*, *Helvia*, and *Theopropus* (ingroup) was compared with the group *Creobroter* (Westwood, 1889), *Chloroharpax* (Werner, 1908), *Panurgica* (Karsch, 1896), *Pseudocreobotra* (Saussure, 1870), and *Chlidonoptera* (Karsch, 1892) (outgroup). In addition, we plotted *Hymenopus* alone to compare with outgroup taxa as well as *Helvia* and *Theopropus* together.

Measurement and ratio data were tested using the PDAP:PDTREE v1.16^60^ module in Mesquite v3.04^61^ by plotting absolute values of the standardised phylogenetically independent contrasts^62^ versus their standard deviations to test if the phylogenetic tree adequately fits the tip data^63^,^64^,^65^. Recovering a least squares regression slope that significantly diverges from zero indicates a poor fit. We tested measurement data using the Hymenopodini-only pruned phylogeny and ratio data (SSD and dimorphism of total proportional femoral leg lobe are) using the extended sampling pruned to Hymenopodinae, Acromantinae, and Oxypilinae taxa (Supplementary Table S10).

Statistical analysis of length measurements, total proportional leg lobe area, and ratios was performed using PAST. Taxa were grouped to assess differences in medians across combinations of taxa using Kruskal-Wallis tests (significance is p < 0.05). Specifically, primary measurement data for body length, pronotum length, and total proportional leg lobe area were tested for both males and females for taxon groups including: 1) (*Hymenopus* + *Helvia* + *Theopropus*) vs. (Hymenopodini outgroup); 2) (*Helvia* + *Theopropus*) vs. (Hymenopodini outgroup); 3) (*Hymenopus*) vs. (Hymenopodini outgroup); and 4) (*Hymenopus*) vs. (*Helvia* + *Theopropus*). Sexual size dimorphism ratio data was grouped including: 1) (*Hymenopus* + *Helvia* + *Theopropus*) vs. (Acromantinae, Oxypilinae, and Hymenopodinae); and 2) (*Helvia* + *Theopropus*) vs. (Acromantinae, Oxypilinae, and Hymenopodinae). These group comparisons targeted the size differences observed between our focal taxa (*Hymenopus*, *Helvia*, *Theopropus*) and the taxa sampled from the rest of Hymenopodinae, Acromantinae, and Oxypilinae. We tested the difference between *Hymenopus* male and female total proportional femoral leg lobe area using the Kruskal-Wallis test.

To test for correlation between male and female pronotum length measurements vs. SSD ratios, we determined the Pearson product-moment correlation coefficient (computed through the origin) and its associated p-value for Y contrasts vs. X contrasts (positivised) as well as Felsenstein’s contrasts^62^. A least squares regression slope that is positive and a p value < 0.05 is considered as support for a correlation between the tested characters.

## Additional Information

**How to cite this article**: Svenson, G. J. *et al*. Selection for predation, not female fecundity, explains sexual size dimorphism in the orchid mantises. *Sci. Rep.* **6**, 37753; doi: 10.1038/srep37753 (2016).

**Publisher's note:** Springer Nature remains neutral with regard to jurisdictional claims in published maps and institutional affiliations.

## Supplementary Material

Supplementary Information
