# Supplementary material for: Selection for predation, not female fecundity, explains sexual size dimorphism in the orchid mantises
Source: Sci Rep. 2016 Dec 1;6:37753. doi: 10.1038/srep37753 (PMC5131372; doi:10.1038/srep37753)
Supplement: Supplementary Information [file srep37753-s1.pdf]

Supplementary Information

Selection for predation, not female fecundity, explains sexual size dimorphism in the orchid mantises

Authors: Gavin J. Svenson[1,2*], Sydney K. Brannoch[1,2], Henrique M. Rodrigues[1,2], James O'Hanlon[3], Frank Wieland[4]

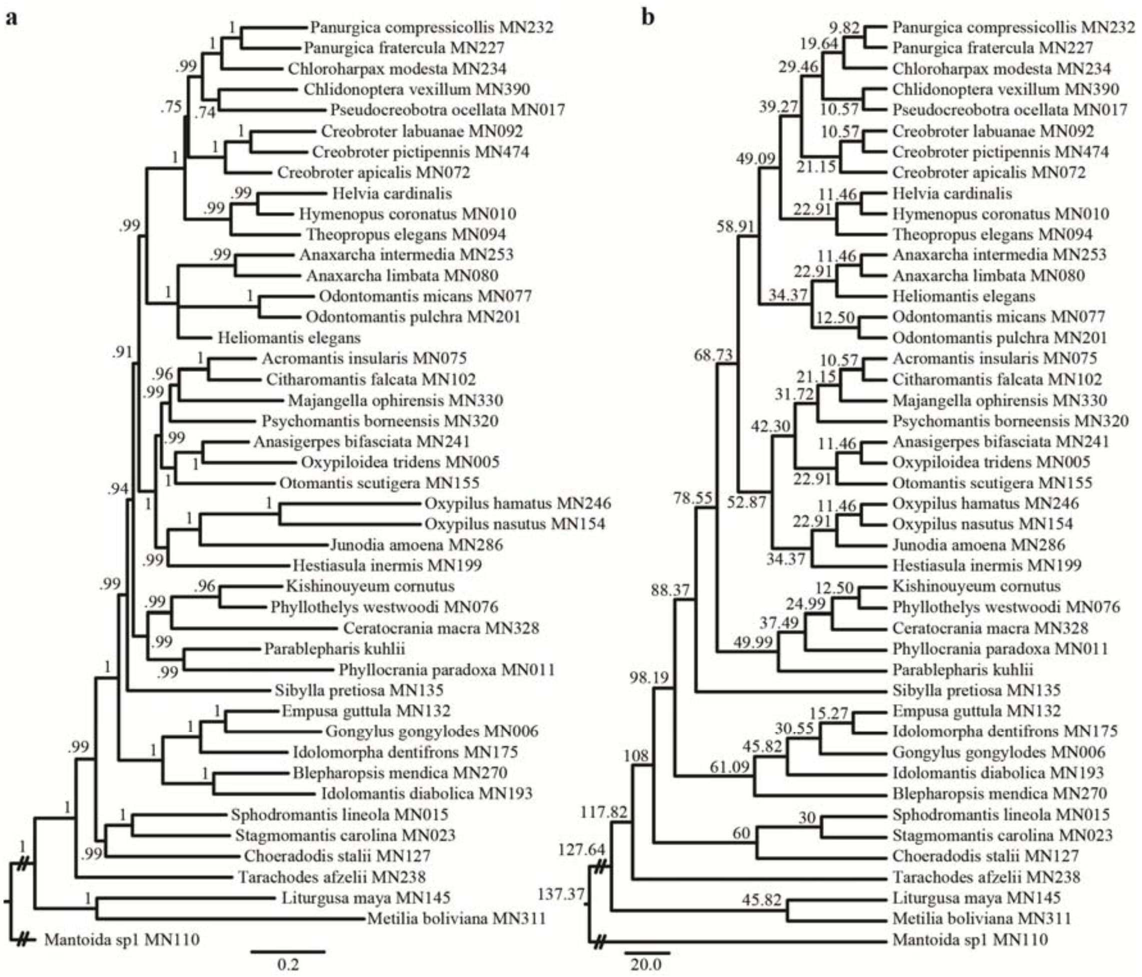

Figure S1. Total evidence analysis phylogenies. (**a**) Mixed model total evidence Bayesian analysis phylogeny (run 1 mean= -6.667057e+004, run 2 mean= -6.666768e+004; harmonic mean=-6.666993e+004) including posterior probabilities for each node. Two deep level branches are not to scale to save space. (**b**) Time calibrated phylogeny estimated with fossil calibration and a root age range taken from prior studies. Average node ages are shown. Two deep level branches are not to scale to save space.

Table S1. Taxon Sampling, specimen voucher codes, and GenBank accession numbers. Genes are abbreviated according to those presented in the Methods. Under Code "morph" is used for taxa that lack molecular data and are included only with morphological data.

**Subfamily**	**Tribe**	**Species**	**Code**	**12S**	**16S**	**18S**	**28S**	**COI**	**COII**	**H2A**	**H3**	**ND4**	**Wnt**
Hymenopodinae	Hymenopodini	*Chlidonoptera vexillum* Karsch, 1892	MN390	-NA-	KR360507	KR360540	KR360577	KR360608	-NA-	-NA-	-NA-	-NA-	KR360712
Hymenopodinae	Hymenopodini	*Chloroharpax modesta* (Gerstaecker, 1883)	MN234	EF383281	EF383439	EF383600	EF383766	EF383920	EF384050	-NA-	EF384177	FJ802633	FJ803089
Hymenopodinae	Hymenopodini	*Creobroter apicalis* Saussure, 1869	MN072	EF383194	EF383354	EF383514	EF383675	EF383837	EF383964	KR360629	EF384093	FJ802487	FJ802985
Hymenopodinae	Hymenopodini	*Creobroter labuanae* Hebard, 1920	MN092	FJ806008	FJ806183	FJ806370	FJ806569	FJ802787	FJ806897	KR360636	FJ806734	FJ802507	FJ803001
Hymenopodinae	Hymenopodini	*Creobroter pictipennis* Wood-Mason, 1878	MN474	KR360496	-NA-	KR360563	KR360596	KR360619	-NA-	-NA-	KR360685	-NA-	KR360718
Hymenopodinae	Hymenopodini	*Helvia cardinalis* Stål, 1877	morph	-NA-	-NA-	-NA-	-NA-	-NA-	-NA-	-NA-	-NA-	-NA-	-NA-
Hymenopodinae	Hymenopodini	*Hymenopus coronatus* (Olivier, 1792)	MN010	EF383156	EF383316	EF383476	EF383638	EF383800	AY491276	KR360626	AY491334	FJ802425	FJ802930
Hymenopodinae	Hymenopodini	*Panurgica compressicollis* Saussure, 1898	MN232	FJ806062	FJ806238	FJ806427	FJ806627	FJ802841	FJ806952	-NA-	FJ806789	FJ802631	FJ803087
Hymenopodinae	Hymenopodini	*Panurgica fratercula* Rehn, 1912	MN227	EF383276	EF383434	EF383595	EF383761	EF383915	EF384046	-NA-	EF384172	FJ802626	FJ803082
Hymenopodinae	Hymenopodini	*Pseudocreobotra ocellata* (P. de Beauvois, 1805)	MN017	FJ805978	FJ806155	FJ806341	FJ806539	FJ802757	AY491283	KR360627	AY491341	FJ802432	FJ802937
Hymenopodinae	Hymenopodini	*Theopropus elegans* (Westwood, 1832)	MN094	FJ806010	FJ806185	FJ806372	FJ806571	FJ802789	FJ806899	KR360637	FJ806735	FJ802509	FJ803003
Hymenopodinae	Anaxarchini	*Anaxarcha intermedia* Mukherjee, 1995	MN253	EF383294	EF383452	EF383613	EF383779	EF383933	EF384063	-NA-	EF384190	FJ802651	FJ803106
Hymenopodinae	Anaxarchini	*Anaxarcha limbata* Giglio-Tos, 1915	MN080	FJ806003	FJ806178	FJ806365	FJ806564	FJ802782	FJ806892	KR360634	FJ806729	FJ802495	FJ802991
Hymenopodinae	Anaxarchini	*Heliomantis elegans* (Navás, 1904)	morph	-NA-	-NA-	-NA-	-NA-	-NA-	-NA-	-NA-	-NA-	-NA-	-NA-
Hymenopodinae	Anaxarchini	*Odontomantis pulchra* (Fabricius, 1787)	MN201	FJ806055	FJ806231	FJ806419	FJ806621	FJ802834	FJ806946	KR360645	FJ806782	FJ802606	-NA-
Hymenopodinae	Anaxarchini	*Odontomantis micans* (Saussure, 1871)	MN077	EF383196	EF383356	EF383516	EF383677	EF383839	EF383966	KR360633	EF384095	FJ802490	FJ802987
Acromantinae	Acromantini	*Acromantis insularis* Giglio-Tos, 1915	MN075	EF383196	EF383356	EF383516	EF383677	EF383839	EF383966	KR360631	EF384095	FJ802490	FJ802987
Acromantinae	Acromantini	*Citharomantis falcata* Rehn, 1909	MN102	EF383212	EF383372	EF383532	EF383695	EF383855	EF383982	-NA-	EF384111	FJ802517	FJ803009
Acromantinae	Acromantini	*Majangella ophirensis* Werner, 1922	MN330	FJ806121	FJ806301	FJ806497	FJ806699	FJ802899	FJ807017	KR360659	FJ806856	FJ802724	FJ803161
Acromantinae	Acromantini	*Psychomantis borneensis* (de Haan, 1842)	MN320	FJ806113	FJ806291	FJ806487	FJ806689	FJ802890	FJ807007	KR360658	FJ806847	FJ802714	FJ803155
Acromantinae	Otomantini	*Anasigerpes bifasciata* Giglio-Tos, 1915	MN241	EF383287	EF383445	EF383606	EF383772	EF383926	EF384056	KR360649	EF384183	FJ802640	FJ803096
Acromantinae	Otomantini	*Otomantis scutigera* Bolivar, 1890	MN155	FJ806036	FJ806211	FJ806398	FJ806597	FJ802816	FJ806925	KR360638	FJ806761	FJ802564	FJ803046
Acromantinae	Otomantini	*Oxypiloidea tridens* (Saussure, 1872)	MN005	FJ805976	FJ806153	FJ806339	FJ806537	FJ802754	AY491273	KR360625	AY491330	FJ802420	FJ802926
Oxypilinae	-	*Hestiasula inermis* (Wood-Mason, 1879)	MN199	EF383257	EF383416	EF383576	EF383742	FJ802833	EF384027	KR360644	EF384154	FJ802604	FJ803067
Oxypilinae	-	*Junodia amoena* Schulthess-Rechberg, 1899	MN286	FJ806086	FJ806260	FJ806452	FJ806650	FJ802861	FJ806975	-NA-	FJ806814	FJ802681	FJ803130
Oxypilinae	-	*Oxypilus hamatus* Roy, 1966	MN246	FJ806066	FJ806242	FJ806431	FJ806631	FJ802845	FJ806956	KR360651	FJ806793	FJ802645	FJ803101
Oxypilinae	-	*Oxypilus nasutus* (Fabricius, 1787)	MN154	FJ806035	FJ806210	FJ806397	FJ806596	FJ802815	FJ806924	-NA-	FJ806760	FJ802563	FJ803045

**Subfamily**	**Tribe**	**Species**	**Code**	**12S**	**16S**	**18S**	**28S**	**COI**	**COII**	**H2A**	**H3**	**ND4**	**Wnt**
Phyllocraniinae	-	*Parablepharis kuhlii* (de Haan, 1842)	morph	-NA-	-NA-	-NA-	-NA-	-NA-	-NA-	-NA-	-NA-	-NA-	-NA-
Phyllocraniinae	-	*Phyllocrania paradoxa* Burmeister, 1838	MN011	EF383157	EF383317	EF383477	EF383639	EF383801	AY491277	-NA-	AY491335	FJ802426	FJ802931
Phyllothelyinae	-	*Ceratocrania macra* Westwood, 1889	MN328	-NA-	FJ806299	FJ806495	FJ806697	FJ802897	FJ807015	-NA-	FJ806854	FJ802722	-NA-
Phyllothelyinae	-	*Kishinouyeum cornutus* Zhang, 1988	morph	-NA-	-NA-	-NA-	-NA-	-NA-	-NA-	-NA-	-NA-	-NA-	-NA-
Phyllothelyinae	-	*Phyllothelys westwoodi* (Wood-Mason, 1876)	MN076	EF383197	EF383357	EF383517	EF383678	EF383840	EF383967	KR360632	EF384096	FJ802491	FJ802988
Sibyllinae	-	*Sibylla pretiosa* Stål, 1856	MN135	FJ806029	FJ806204	FJ806391	FJ806590	FJ802808	FJ806918	-NA-	FJ806754	FJ802548	FJ803032
Blepharodinae	Blepharodini	*Blepharopsis mendica* (Fabricius, 1775)	MN270	FJ806077	-NA-	FJ806442	-NA-	-NA-	FJ806966	KR360652	FJ806803	FJ802666	FJ803117
Blepharodinae	Idolomantini	*Idolomantis diabolica* (Saussure, 1869)	MN193	EF383253	EF383412	EF383572	EF383738	EF383894	EF384023	KR360643	EF384150	FJ802598	FJ803061
Empusinae	Empusini	*Empusa guttula* (Thunberg, 1815)	MN132	EF383226	EF383386	EF383546	EF383709	EF383869	EF383996	-NA-	EF384125	FJ802545	FJ803029
Empusinae	Empusini	*Gongylus gongylodes* (Linneaus, 1758)	MN006	EF383152	EF383312	EF383472	EF383634	EF383797	AY491274	-NA-	AY491331	FJ802421	-NA-
Empusinae	Idolomorphini	*I. dentifrons* Saussure & Zehntner, 1895	MN175	EF383246	EF383405	EF383565	EF383731	EF383887	EF384016	KR360642	EF384143	FJ802581	FJ803057
-	-	*Mantoida* sp.	MN110	FJ806015	FJ806190	FJ806377	FJ806576	FJ802794	FJ806904	-NA-	FJ806740	FJ802524	-NA-
-	-	*Liturgusa maya* Saussure & Zehntner, 1894	MN145	EF383231	EF383391	EF383551	EF383714	FJ802812	EF384001	-NA-	EF384130	FJ802555	FJ803037
Acanthopinae	-	*Metilia boliviana* (Werner, 1927)	MN311	FJ806107	FJ806282	FJ806478	FJ806680	FJ802881	FJ806999	KR360657	FJ806838	FJ802705	FJ803149
Choeradodinae	-	*Choeradodis stalii* Wood-Mason, 1880	MN127	EF383225	EF383385	EF383545	EF383708	EF383868	EF383995	-NA-	EF384124	FJ802540	FJ803024
Mantinae	-	*Sphodromantis lineola* (Burmeister, 1838)	MN015	EF383160	EF383320	EF383480	EF383642	EF383804	AY491281	-NA-	AY491339	FJ802430	FJ802935
Stagmomantinae	-	*Stagmomantis carolina* (Johansson, 1763)	MN023	EF383165	EF383325	EF383485	EF383647	EF383809	AY491289	-NA-	AY491347	FJ802438	FJ802943
Tarachodinae	-	*Tarachodes afzelii* (Stål, 1871)	MN238	EF383285	EF383443	EF383604	EF383770	EF383924	EF384054	-NA-	EF384181	FJ802637	FJ803093

Table S2. Complete measurement data for all 129 measured individuals across the taxon sampling. Body length, pronotum length, meso- and metafemoral lobe apical lobe area, and meso- and metafemoral length are all direct measurements. Two ratios are present with an additive total category.

Taxon	Sex	Specimen	Body Length	Pronotum Length	Mesofemoral ventral apical lobe area	Metafemoral ventral apical lobe area	Mesofemoral Length	Metafemoral Length	Size ratio of mesofemoral leg lobe area	Size ratio of metathoracic leg lobe area	Total Prop Leg Lobe Area
Creobroter pictipennis	Male	GSMC003546	22.859	5.453	0.190	0.587	5.644	6.632	0.034	0.089	0.063
Creobroter pictipennis	Male	GSMC003909	27.817	6.050	0.142	0.477	6.553	7.330	0.022	0.065	0.045
Creobroter pictipennis	Male	GSMC003983	29.133	6.248	0.192	0.552	7.084	7.997	0.027	0.069	0.049
Creobroter pictipennis	Female	GSMC003984	29.207	7.145	0.382	0.995	6.538	7.933	0.058	0.125	0.095
Creobroter pictipennis	Female	MANTO0002	27.446	7.111	0.302	0.897	6.642	7.659	0.045	0.117	0.084
Creobroter pictipennis	Female	GSMC003545	28.984	6.689	0.343	0.836	6.517	7.834	0.053	0.107	0.082
Creobroter apicalis	Male	GSMC002236	24.993	6.680	0.915	1.687	7.812	8.853	0.117	0.191	0.156
Creobroter apicalis	Male	GSMC002225	25.943	6.357	0.529	1.141	6.973	7.760	0.076	0.147	0.113
Creobroter apicalis	Female	GSMC002210	33.523	9.353	1.750	2.993	9.275	10.809	0.189	0.277	0.236
Creobroter apicalis	Female	GSMC002227	31.892	8.505	1.215	2.075	8.656	10.094	0.140	0.206	0.175
Creobroter labuanae	Male	USNM00873192	28.748	6.102	0.394	0.909	7.084	8.003	0.056	0.114	0.086
Creobroter labuanae	Male	USNM00873199	27.713	6.218	0.426	0.658	7.195	8.039	0.059	0.082	0.071
Creobroter labuanae	Male	USNM00873200	27.900	6.423	0.389	0.734	7.259	8.041	0.054	0.091	0.073
Creobroter labuanae	Male	USNM00873206	27.496	6.251	0.334	0.780	6.938	8.040	0.048	0.097	0.074
Creobroter labuanae	Male	USNM00873209	28.454	6.401	0.308	0.816	7.461	8.557	0.041	0.095	0.070
Creobroter labuanae	Female	USNM00873204	35.781	8.290	1.385	2.552	8.161	9.520	0.170	0.268	0.223
Creobroter labuanae	Female	USNM00873230	31.152	8.083	1.446	2.126	8.167	9.554	0.177	0.223	0.202
Creobroter labuanae	Female	USNM00873231	36.790	8.230	1.231	2.105	8.190	9.545	0.150	0.221	0.188
Creobroter labuanae	Female	USNM00873234	29.617	7.169	1.071	1.938	7.078	8.332	0.151	0.233	0.195
Creobroter labuanae	Female	Mindanao	38.018	8.127	1.289	2.656	8.280	9.509	0.156	0.279	0.222
Panurgica fratercula	Male	GSMC000825	22.532	4.857	0.404	0.838	5.997	6.720	0.067	0.125	0.098
Panurgica fratercula	Male	USNMENT00873129	23.490	4.815	0.274	0.594	5.780	6.538	0.047	0.091	0.070
Panurgica fratercula	Female	GSMC003481	26.256	6.259	1.196	2.073	5.917	7.114	0.202	0.291	0.251

Taxon	Sex	Specimen	Body Length	Pronotum Length	Mesofemoral ventral apical lobe area	Metafemoral ventral apical lobe area	Mesofemoral Length	Metafemoral Length	Size ratio of mesofemoral leg lobe area	Size ratio of metathoracic leg lobe area	Total Prop Leg Lobe Area
Panurgica compressicollis	Male	GSMC004158	30.266	5.588	0.847	1.356	6.223	7.202	0.136	0.188	0.164
Panurgica compressicollis	Male	GSMC003520	28.070	6.001	0.841	1.258	6.555	7.427	0.128	0.169	0.150
Panurgica compressicollis	Male	Liberia	27.210	5.548	0.452	0.707	7.775	7.120	0.058	0.099	0.078
Panurgica compressicollis	Female	B. Congo	40.067	7.511	2.425	3.028	7.162	8.296	0.339	0.365	0.353
Panurgica compressicollis	Female	B. Congo	38.049	7.064	2.234	3.429	6.740	7.919	0.331	0.433	0.386
Chloroharpax modesta	Male	GSMC003512	26.328	5.434	0.099	0.070	5.706	5.947	0.017	0.012	0.015
Chloroharpax modesta	Male	GSMC004241	23.581	4.780	0.000	0.000	5.313	5.514	0.000	0.000	0.000
Chloroharpax modesta	Male	GSMC003514	23.299	4.749	0.000	0.037	5.166	5.619	0.000	0.007	0.003
Chloroharpax modesta	Male	GSMC004124	21.786	4.555	0.323	0.094	4.760	5.374	0.068	0.017	0.041
Chloroharpax modesta	Male	GSMC003513	27.607	5.300	0.248	0.086	5.693	6.202	0.044	0.014	0.028
Chloroharpax modesta	Female	Liberia	30.464	6.664	0.092	0.200	6.612	7.330	0.014	0.027	0.021
Chloroharpax modesta	Female	Liberia	31.023	6.555	0.026	0.085	6.423	6.996	0.004	0.012	0.008
Chloroharpax modesta	Female	USNMENT00873161	27.336	6.675	0.000	0.000	7.042	6.997	0.000	0.000	0.000
Chloroharpax modesta	Female	USNMENT00873162	26.000	6.517	0.000	0.000	6.121	6.920	0.000	0.000	0.000
Pseudocreobotra ocellata	Male	GSMC005119	24.369	4.573	0.833	1.180	5.961	6.763	0.140	0.174	0.158
Pseudocreobotra ocellata	Male	Liberia, Monrovia, VI-22-1958	23.448	5.144	0.927	1.268	6.606	7.478	0.140	0.170	0.156
Pseudocreobotra ocellata	Male	Angola, Saurimo, 1950	29.239	5.133	1.853	2.685	6.863	7.763	0.270	0.346	0.310
Pseudocreobotra ocellata	Male	GSMC002176	25.606	5.040	1.762	2.398	6.918	7.399	0.255	0.324	0.291
Pseudocreobotra ocellata	Male	Garua	26.290	4.629	1.255	1.154	6.175	7.111	0.203	0.162	0.181
Pseudocreobotra ocellata	Female	GSMC005120	33.274	6.625	2.280	2.741	7.524	8.703	0.303	0.315	0.309
Pseudocreobotra ocellata	Female	GSMC004486	29.103	5.551	1.541	2.484	6.766	8.080	0.228	0.307	0.271
Pseudocreobotra ocellata	Female	Mali	27.751	5.594	1.923	3.110	6.600	7.775	0.291	0.400	0.350
Chlidonoptera lestoni	Male	GSMC004222	32.227	6.211	0.879	1.454	6.884	8.583	0.128	0.169	0.151
Chlidonoptera lestoni	Male	GSMC004180	31.282	6.475	1.223	1.615	7.599	8.620	0.161	0.187	0.175
Chlidonoptera lestoni	Male	GSMC004240	28.972	6.131	1.075	1.499	7.456	8.345	0.144	0.180	0.163
Chlidonoptera lestoni	Male	GSMC004221	30.331	6.331	1.224	1.660	7.616	8.599	0.161	0.193	0.178
Chlidonoptera lestoni	Male	GSMC004188	27.150	5.968	0.845	1.353	7.164	7.930	0.118	0.171	0.146
Chlidonoptera lestoni	Male	GSMC004187	28.344	6.429	1.321	1.667	7.342	8.430	0.180	0.198	0.189
Chlidonoptera lestoni	Male	Wien117	28.637	6.145	1.300	1.841	7.231	8.159	0.180	0.226	0.204
Chlidonoptera lestoni	Male	Wien116	29.004	6.391	1.500	1.775	7.521	8.488	0.199	0.209	0.205
Chlidonoptera lestoni	Male	Wien118	27.097	6.149	1.340	1.900	7.220	8.138	0.186	0.233	0.211

Taxon	Sex	Specimen	Body Length	Pronotum Length	Mesofemoral ventral apical lobe area	Metafemoral ventral apical lobe area	Mesofemoral Length	Metafemoral Length	Size ratio of mesofemoral leg lobe area	Size ratio of metathoracic leg lobe area	Total Prop Leg Lobe Area
Chlidonoptera lestoni	Female	Wien119	33.203	8.391	5.080	6.159	8.900	10.055	0.571	0.613	0.593
Hymenopus coronatus	Male	GSMC003908	28.258	5.739	6.788	7.477	6.578	7.563	1.032	0.989	1.009
Hymenopus coronatus	Male	GSMC003549	28.649	5.984	8.236	8.295	6.591	7.636	1.250	1.086	1.162
Hymenopus coronatus	Male	GSMC003548	27.884	5.955	8.748	8.365	7.058	7.926	1.239	1.055	1.142
Hymenopus coronatus	Male	GSMC003550	28.622	6.009	8.009	6.385	7.092	7.183	1.129	0.889	1.008
Hymenopus coronatus	Male	GSMC003551	27.106	5.834	9.478	10.140	7.148	8.115	1.326	1.250	1.285
Hymenopus coronatus	Female	USNMENT01073092	56.567	14.260	71.887	86.681	14.343	16.681	5.012	5.196	5.111
Hymenopus coronatus	Female	USNMENT01073112	54.625	12.888	57.865	68.733	13.222	14.922	4.376	4.606	4.498
Hymenopus coronatus	Female	Depol	50.594	12.072	49.926	59.095	12.251	14.230	4.075	4.153	4.117
Hymenopus coronatus	Female	USNMENT01073243	52.112	12.998	50.416	55.965	12.821	14.388	3.932	3.890	3.910
Helvia cardinalis	Male	GSMC003552	23.547	4.768	0.864	1.244	4.766	5.323	0.181	0.234	0.209
Helvia cardinalis	Male	Holotype NRM-MANT 0002869	14.914	3.518	0.700	1.100	3.179	3.600	0.220	0.306	0.266
Helvia cardinalis	Male	Cameron Highlands, SMNK	19.960	4.475	-	-	-	-	-	-	-
Helvia cardinalis	Female	Trong	42.729	9.261	7.107	8.211	8.265	9.127	0.860	0.900	0.881
Helvia cardinalis	Female	Cameron Highlands, SMNK	32.030	8.250	-	-	-	-	-	-	-
Theopropus elegans	Male	Borneo, Crocker Range	20.138	4.661	0.476	0.693	4.283	5.118	0.111	0.135	0.124
Theopropus elegans	Male	Sabah BMNH	20.230	4.624	0.359	0.597	4.328	4.865	0.083	0.123	0.104
Theopropus elegans	Male	Sabah BMNH	20.873	4.657	0.468	0.577	4.261	5.065	0.110	0.114	0.112
Theopropus elegans	Male	USNMENT01073151	19.627	4.436	0.461	0.714	4.417	4.943	0.104	0.144	0.126
Theopropus elegans	Female	USNMENT01073154	40.678	10.231	2.528	3.637	9.745	10.942	0.259	0.332	0.298
Theopropus elegans	Female	GSMC002149	38.340	10.920	3.005	3.212	9.702	11.318	0.310	0.284	0.296
Theopropus elegans	Female	GSMC003791	37.429	9.661	2.508	3.706	9.166	10.761	0.274	0.344	0.312
Theopropus elegans	Female	Malaysia, KL	39.693	10.802	3.105	4.264	9.638	11.241	0.322	0.379	0.353
Theopropus elegans	Female	Sumatra	43.899	10.581	2.734	4.947	10.293	11.967	0.266	0.413	0.345
Anaxarcha intermedia	Male	GSMC002237	23.500	7.705	0.000	0.000	6.000	6.661	0.000	0.000	0.000
Anaxarcha intermedia	Female	India: Kerala, BMNH	32.404	10.076	0.000	0.000	7.099	7.648	0.000	0.000	0.000
Anaxarcha limbata	Male	GSMC004558	29.134	10.089	0.000	0.000	7.569	8.973	0.000	0.000	0.000
Anaxarcha limbata	Female	GSMC004559	39.296	12.185	0.000	0.000	8.991	10.626	0.000	0.000	0.000
Anaxarcha limbata	Female	GSMC004560	39.493	12.858	0.000	0.000	8.778	10.392	0.000	0.000	0.000
Heliomantis elegans	Male	BMNH	34.000	11.500	0.100	0.400	7.550	9.100	0.013	0.044	0.030
Heliomantis elegans	Female	BMNH	47.000	15.000	0.210	0.610	10.790	12.930	0.019	0.047	0.035
Odontomantis pulchra	Male	Taiwan, CASENT	17.082	5.010	0.000	0.000	4.415	5.210	0.000	0.000	0.000

Taxon	Sex	Specimen	Body Length	Pronotum Length	Mesofemoral ventral apical lobe area	Metafemoral ventral apical lobe area	Mesofemoral Length	Metafemoral Length	Size ratio of mesofemoral leg lobe area	Size ratio of metathoracic leg lobe area	Total Prop Leg Lobe Area
Odontomantis pulchra	Male	Taiwan, CASENT	17.427	4.766	0.000	0.000	3.952	5.203	0.000	0.000	0.000
Odontomantis pulchra	Female	Taiwan, CASENT	24.068	6.450	0.000	0.000	5.493	6.477	0.000	0.000	0.000
Odontomantis pulchra	Female	Taiwan, CASENT	22.502	6.469	0.000	0.000	5.403	6.368	0.000	0.000	0.000
Odontomantis pulchra	Female	Taiwan, CASENT	22.299	6.368	0.000	0.000	5.659	6.552	0.000	0.000	0.000
Odontomantis micans	Male	GSMC000751	14.212	4.169	0.000	0.000	3.790	4.278	0.000	0.000	0.000
Odontomantis micans	Male	GSMC000749	13.895	4.382	0.000	0.000	4.005	4.691	0.000	0.000	0.000
Odontomantis micans	Female	GSMC000748	18.900	5.322	0.000	0.000	4.713	5.471	0.000	0.000	0.000
Acromantis insularis	Female	GSMC002214	30.287	10.690	0.841	1.463	6.082	7.672	0.138	0.191	0.168
Acromantis insularis	Male	GSMC002209	24.770	9.072	0.272	0.495	5.294	6.553	0.051	0.076	0.065
Citharomantis falcata	Male	GSMC002196	20.105	6.494	0.045	0.066	4.820	5.482	0.009	0.012	0.011
Citharomantis falcata	Male	Borneo	21.647	6.407	0.066	0.072	5.231	5.978	0.013	0.012	0.012
Citharomantis falcata	Female	GSMC003538	29.823	8.875	0.259	0.327	6.357	7.412	0.041	0.044	0.043
Citharomantis falcata	Female	CASENT Malaysia	27.592	9.095	0.237	0.339	6.448	7.664	0.037	0.044	0.041
Majangella ophirensis	Female	GSMC005128	37.200	11.946	0.151	0.216	8.744	9.548	0.017	0.023	0.020
Majangella ophirensis	Male	GSMC005127	30.179	9.431	0.053	0.078	7.141	8.382	0.007	0.009	0.008
Majangella ophirensis	Male	GSMC003553	33.064	9.165	0.170	0.238	7.100	8.389	0.024	0.028	0.026
Majangella ophirensis	Male	GSMC003554	31.371	8.797	0.185	0.213	7.119	8.395	0.026	0.025	0.026
Psychomantis borneensis	Female	GSMC003985	42.347	14.274	0.888	1.180	7.154	9.465	0.124	0.125	0.124
Psychomantis borneensis	Female	GSMC003490	42.055	14.700	0.795	0.888	7.503	9.053	0.106	0.098	0.102
Psychomantis borneensis	Male	GSMC003903	33.761	11.903	0.396	0.568	5.944	7.736	0.067	0.073	0.070
Psychomantis borneensis	Male	GSMC002148	31.155	12.356	0.608	0.641	6.329	7.939	0.096	0.081	0.088
Anasigerpes bifasciata	Female	GSMC003537	22.573	6.751	0.222	0.312	4.058	5.292	0.055	0.059	0.057
Anasigerpes bifasciata	Female	GSMC004531	24.652	6.760	0.181	0.299	4.005	5.202	0.045	0.057	0.052
Anasigerpes bifasciata	Male	GSMC003553	18.613	5.606	0.096	0.093	3.598	4.646	0.027	0.020	0.023
Anasigerpes bifasciata	Male	GSMC004128	18.728	5.369	0.082	0.065	3.489	4.408	0.024	0.015	0.019
Oxypiloidea tridens	Male	GSMC002107	19.941	5.564	0.307	0.254	2.944	4.200	0.104	0.060	0.079
Oxypiloidea tridens	Female	GSMC002258	23.690	7.375	0.456	0.329	3.183	4.477	0.143	0.073	0.102
Otomantis scutigera	Male	B.Congo	20.150	4.200	0.044	0.129	4.044	5.160	0.011	0.025	0.019
Otomantis scutigera	Male	Lombardo specimen	17.000	4.300	-	-	-	-	-	-	-
Otomantis scutigera	Female	Lombardo specimen	22.500	5.800	-	-	-	-	-	-	-
Oxypilus hamatus	Male	Nigeria CASENT	16.046	3.414	0.000	0.000	4.000	4.200	0.000	0.000	0.000
Oxypilus hamatus	Male	Nigeria CASENT	14.870	3.164	0.000	0.000	4.100	4.300	0.000	0.000	0.000
Oxypilus hamatus	Female	Nigeria CASENT	14.500	4.100	0.000	0.000	5.000	5.000	0.000	0.000	0.000

Taxon	Sex	Specimen	Body Length	Pronotum Length	Mesofemoral ventral apical lobe area	Metafemoral ventral apical lobe area	Mesofemoral Length	Metafemoral Length	Size ratio of mesofemoral leg lobe area	Size ratio of metathoracic leg lobe area	Total Prop Leg Lobe Area
Oxypilus nasutus	Female	GSMC002189	15.388	5.213	0.000	0.000	4.306	5.173	0.000	0.000	0.000
Oxypilus nasutus	Male	GSMC002188	18.983	4.081	0.000	0.000	4.540	5.167	0.000	0.000	0.000
Oxypilus nasutus	Male	GSMC002187	21.378	4.550	0.000	0.000	4.500	5.000	0.000	0.000	0.000
Junodia amoena	Male	GSMC002177	15.852	2.750	0.047	0.070	3.962	4.124	0.012	0.017	0.014
Junodia amoena	Female	Malawi, Carnegie	17.022	3.700	0.101	0.097	3.728	4.067	0.027	0.024	0.025
Hestiasula inermis	Male	CMNH	26.367	4.784	0.000	0.000	5.402	6.147	0.000	0.000	0.000
Hestiasula inermis	Male	CMNH	25.887	4.429	-	-	-	-	-	-	-
Hestiasula inermis	Male	CMNH	-	4.479	-	-	-	-	-	-	-
Hestiasula inermis	Male	CMNH	25.211	4.582	-	-	-	-	-	-	-
Hestiasula inermis	Female	CMNH	27.149	5.932	0.000	0.000	5.494	6.475	0.000	0.000	0.000

Table S3. Summarized measurement data for Hymenopodini. Average measurements by sex for body length, pronotum length, and total proportional femoral leg lobe area. Standard deviations are included within parentheses. *only a single female specimen was obtained for measurements.

Taxon	Male body length	Female body length	Male pronotum length	Female pronotum length	Male total proportional femoral leg lobe area	Female total proportional femoral leg lobe area
Creobroter pictipennis	26.60 (3.31)	28.60 (0.96)	5.92 (0.41)	6.98 (0.25)	0.05 (0.01)	0.09 (0.01)
Creobroter apicalis	25.47 (0.67)	32.71 (1.15)	6.52 (0.23)	8.93 (0.60)	0.13 (0.03)	0.21 (0.04)
Creobroter labuanae	28.06 (0.52)	34.27 (3.68)	6.28 (0.13)	7.98 (0.46)	0.08 (0.01)	0.21 (0.02)
*Panurgica fratercula**	23.01 (0.68)	26.26 (-)	4.84 (0.03)	6.26 (-)	0.08 (0.02)	0.25 (-)
Panurgica compressicollis	28.52 (1.58)	39.06 (1.43)	5.71 (0.25)	7.29 (0.32)	0.13 (0.05)	0.37 (0.02)
Chloroharpax modesta	24.52 (2.38)	28.71 (2.43)	4.96 (0.38)	6.60 (0.08)	0.02 (0.02)	0.01 (0.01)
Pseudocreobotra ocellata	25.79 (2.22)	30.04 (2.88)	4.90 (0.28)	5.92 (0.61)	0.22 (0.08)	0.31 (0.04)
*Chlidonoptera lestoni**	29.23 (1.73)	33.20 (-)	6.25 (0.17)	8.39 (-)	0.18 (0.02)	0.59 (-)
Hymenopus coronatus	28.10 (0.64)	53.47 (2.65)	5.90 (0.11)	13.05 (0.90)	1.12 (0.12)	4.41 (0.53)
Helvia cardinalis	19.47 (4.34)	37.38 (7.57)	4.25 (0.65)	8.76 (0.71)	0.24 (0.04)	0.88 (-)
Theopropus elegans	20.22 (0.51)	40.01 (2.51)	4.59 (0.11)	10.44 (0.51)	0.12 (0.01)	0.32 (0.03)

Table S4. Sexual size dimorphism ratios for HOA taxa. Size dimorphism for Hymenopodinae, Oxypilinae, and Acromantinae taxa represented as a female over male ratio for pronotum length and total proportional femoral leg lobe area.

Subfamily	Taxon	Pronotum length dimorphism	Total proportional femoral leg lobe area dimorphism
Hymenopodinae	*Creobroter pictipennis*	1.07	1.66
Hymenopodinae	*Creobroter apicalis*	1.28	1.53
Hymenopodinae	*Creobroter labuanae*	1.22	2.74
Hymenopodinae	*Panurgica fratercula*	1.14	2.98
Hymenopodinae	*Panurgica compressicollis*	1.37	2.83
Hymenopodinae	*Chloroharpax modesta*	1.17	0.42
Hymenopodinae	*Pseudocreobotra ocellata*	1.16	1.41
Hymenopodinae	*Chlidonoptera lestoni*	1.14	3.29
Hymenopodinae	*Hymenopus coronatus*	1.90	3.93
Hymenopodinae	*Helvia cardinalis*	1.92	3.71
Hymenopodinae	*Theopropus elegans*	1.98	2.75
Hymenopodinae	*Anaxarcha intermedia*	1.38	0.00
Hymenopodinae	*Anaxarcha limbata*	1.35	0.00
Hymenopodinae	*Heliomantis elegans*	1.38	0.00
Hymenopodinae	*Odontomantis pulchra*	1.33	0.00
Hymenopodinae	*Odontomantis micans*	1.34	0.00
Acromantinae	*Acromantis insularis*	1.22	2.59
Acromantinae	*Citharomantis falcata*	1.38	3.61
Acromantinae	*Majangella ophirensis*	1.18	1.00
Acromantinae	*Psychomantis borneensis*	1.30	1.43
Acromantinae	*Anasigerpes bifasciata*	1.26	2.63
Acromantinae	*Oxypiloidea tridens*	1.19	1.31
Acromantinae	*Otomantis scutigera*	1.21	2.00
Oxypilinae	*Oxypilus hamatus*	0.94	0.00
Oxypilinae	*Oxypilus nasutus*	0.76	0.00
Oxypilinae	*Junodia amoena*	1.07	1.76
Oxypilinae	*Hestiasula inermis*	1.05	0.00

Table S5. Kruskal-Wallis tests on trait data for Hymenopodini. Male pronotum length, female pronotum length, and male and female proportional leg lobe area were tested for five groupings across the tribe between and within THH taxa and other Hymenopodini taxa.

Group tests	Male pronotum length	Female pronotum length	Male total proportional femoral leg lobe area	Female total proportional femoral leg lobe area
THH clade vs. Outgroup Hymenopodini	0.00434700	0.00001164	0.00292000	0.00034860
(*Theopropus* + *Helvia*) vs. Outgroup Hymenopodini	0.00020950	0.00027870	0.29650000	0.01298000
Hymenopus vs. Outgroup Hymenopodini	0.76870000	0.00185000	0.00034460	0.00183500
Hymenopus vs. (Outgroup Hymenopodini + *Helvia* + *Theopropus*)	0.71100000	0.00141800	0.00029740	0.00143900
Hymenopus vs. (*Theopropus* + *Helvia*)	0.00448300	0.00815100	0.00605400	0.01005000

Table S6. Kruskal-Wallis tests on SSD ratios across HOA taxa. Two group tests between THH (including and excluding *Hymenopus*) and HOA taxa were performed for SSD ratios of pronotum length, and total proportional femoral leg lobe area.

Group tests	Pronotum length dimorphism	Total proportional femoral leg lobe area dimorphism
THH clade vs. HOA clade	0.005436	0.012380
(*Theopropus* + *Helvia*) vs. HOA clade	0.020790	0.050850

Table S7. Correlation of SSD ratios with male and female size. Values of pronotum length tested against pronotum SSD ratios for males and females including Pearson product-moment correlation coefficient p-value for Y contrasts vs. X contrasts (positivised) as well as Felsenstein's contrasts.

Correlation tests	Felsenstein's Contrasts (p-vaule)	Positivised Contrasts (2-tailed p-vaule)	Positivised Contrasts (1-tailed p-vaule)	Positivised Contrasts (Least Squares Regression Slope)
Male pronotum length vs. Pronotum length dimorphism	0.205148	0.4102972	0.205148	-0.5117949
Female pronotum length vs. Pronotum length dimorphism	0.0010624	0.002124	0.00106	4.346948

Table S8. Posterior model distribution. Bayesian analysis of macroevolutionary mixtures posterior distribution represented as percentages for rate shift models for trait data and SSD ratios.

Rate shift model	Pronotum length dimorphism	Total proportional femoral leg lobe area dimorphism	Male pronotum length	Female pronotum length	Male total proportional femoral leg lobe area	Female total proportional femoral leg lobe area
0	2.0%	0.0%	49.0%	41.0%	0.0%	0.0%
1	50.0%	12.0%	29.0%	32.0%	71.0%	70.0%
2	30.0%	9.6%	14.0%	17.0%	23.0%	24.0%
3	12.0%	42.0%	5.5%	6.7%	5.3%	5.6%
4	3.6%	24.0%	1.8%	2.2%	1.0%	1.0%
5	0.9%	8.8%	0.6%	0.6%	0.2%	0.2%
6	0.2%	2.4%	0.1%	0.2%	0.0%	0.0%
7	0.0%	0.6%	0.0%	0.0%	0.0%	0.0%
8	0.0%	0.1%	0.0%	0.0%	0.0%	0.0%
9	0.0%	0.0%	0.0%	0.0%	0.0%	0.0%

Table S9. Shift model support. Bayes factors of rate shift models relative to the null shift model in Bayesian analysis of macroevolutionary mixtures for trait data and SSD ratios.

Rate shift model	Pronotum length dimorphism	Total proportional femoral leg lobe area dimorphism	Male pronotum length	Female pronotum length	Male total proportional femoral leg lobe area	Female total proportional femoral leg lobe area
0	1.00	0.00	1.00	1.00	1.00	1.00
1	25.25	1.00	0.88	1.13	2287.01	8352.47
2	29.41	1.49	0.83	1.16	1402.64	5470.12
3	27.74	15.69	0.77	1.11	790.85	3132.96
4	24.04	26.38	0.74	1.06	428.48	1666.43
5	20.47	31.51	0.76	0.96	223.90	1029.89
6	16.32	31.75	0.63	0.96	141.97	658.78
7	13.78	29.05	0.77	0.91	81.32	346.41
8	13.84	24.47	0.69	1.03	49.27	367.31
9	3.58	17.85	0.22	0.50	0.00	0.00

Table S10. Trait data and ratios fit to phylogeny. Phylogenetic independent contrasts testing fit of tip data to tree for measurements (Hymenopodini taxa only) and ratios (HOA clade). Plots of absolute values of the standardized phylogenetically independent contrasts versus their standard deviations provided least-squares linear regression slopes, none of which differed significantly from zero ($p < 0.05$) indicating that we can reject a tree exhibiting significant lack of fit to tip data.

PIC of Traits	Male pronotum length	Female pronotum length	Male total proportional femoral leg lobe area	Female total proportional femoral leg lobe area	Body length dimorphism	Pronotum length dimorphism	Total proportional femoral leg lobe area dimorphism
Data Points	10	10	10	10	26	26	26
F statistic	0.07208	0.03832	0.36202	0.27882	0.00002	0.37961	2.31415
t statistics	-0.26848	-0.19575	-0.60168	-0.52803	0.00469	0.61613	-1.52123
d.f	8	8	8	8	24	24	24
p value - 2 tailed	0.79512	0.84969	0.56405	0.61181	0.99630	0.54361	0.14127
p vaule - 1 tailed	0.39756	0.42484	0.28202	0.30590	0.49815	0.27181	0.07063
Least Squares Regression Slope	-1.58964	-3.61249	-1.92164	-7.14195	0.00209	0.31973	-3.32829
